# Non-lupus full-house nephropathy—immune dysregulation as a rare cause of pediatric nephrotic syndrome: Answers

**DOI:** 10.1007/s00467-021-05378-0

**Published:** 2021-12-17

**Authors:** Orsolya Horváth, György S. Reusz, Veronika Goda, Kata Kelen, István Balogh, Magdolna Kardos, Krisztián Kállay, Áron Cseh, Attila J. Szabó, Gergely Kriván

**Affiliations:** 1 1St Department of Pediatrics, Semmelweis University, 53-54 Bókay János Street, Budapest, 1083 Hungary; 2 Pediatric Hematology and Stem Cell Transplantation Unit, Central Hospital of Southern Pest, National Institute of Hematology and Infectious Diseases, Budapest, Hungary; 3 Department of Laboratory Medicine, Division of Clinical Genetics, Department of Human Genetics, Faculty of Medicine, University of Debrecen, Debrecen, Hungary; 4 2Nd Department of Pathology, Semmelweis University, Budapest, Hungary

**Keywords:** “Full-house” nephropathy (FHN), Autoimmunity, Primary immunodeficiency (PID), IPEX syndrome, *FOXP3* mutation

## 1. What is the most likely diagnosis?

Based on the synchroneity of severe infections, multiple autoimmune disorders, atopic dermatitis, chronic diarrhoea and insufficient weight gain, the “immunodysregulation, polyendocrinopathy, enteropathy, X-linked” (IPEX) or IPEX-like syndrome was first suspected at the age of 8 in 2015, and calcineurin inhibitor tacrolimus was introduced [1].

IPEX syndrome is a rare primary immunodeficiency syndrome characterized by the development of multiple autoimmune disorders. IPEX is caused by mutations in the forkhead box protein 3 gene (*FOXP3*), which encodes a key transcription factor required for regulatory T cell (Treg) development, maintenance and function [2]. In addition to the traditional clinical presentation (severe enteropathy, type 1 diabetes and skin lesions), IPEX may include other variable and distinct clinical manifestations [3]. The coding region of *FOXP3* was analysed by Sanger sequencing and a silent mutation was detected (c.816G > A). To assess the potential effect of the mutation on RNA splicing, RNA of peripheral white blood cells was tested and the skipping of exon 7 was observed; thus, the effect of the mutation was p.Leu246fs*160 [4].

Treg cell dysfunction is the main pathogenic event leading to multiorgan autoimmunity in IPEX [3]. Functional data demonstrate that Treg cells isolated from IPEX patients are dysfunctional, as they cannot inhibit proliferation and cytokine production of autologous or allogenic T effector cells [3].

Our patient had typical (enteropathy, skin lesions, severe infections) and atypical (non-lupus full-house nephropathy) symptoms without type 1 diabetes leading to the diagnosis of IPEX. Treg cell count was at the lower limit of normal range (CD3 + /CD4 + T cell count 503 cell/μL (lower limit of normal 300 cell/μL), 4.6% Treg cells of the CD3 + /CD4 + cells (normal range 4–9%)). Treg cell subpopulations and intracellular cytokine production were not evaluated and functional T cell tests were not available at the time of diagnosis.

## 2. What is the possible underlying mechanism of kidney involvement?

Non-lupus full-house nephropathy (FHN) was the most remarkable and rare symptom of the multiple autoimmune disorder. FHN is a rare kidney disease characterized by a variety of glomerular lesions typically accompanied by extensive deposition of all classes of immunoglobulins and complement components along different sites of the glomerulus [5]. Different forms of kidney injury have been reported in IPEX syndrome, such as membranous nephropathy and minimal change nephrotic syndrome [6–8]. B cells in IPEX show altered antibody production, and tissue-specific autoantibodies can be detected early [9]. Anti-villin and harmonin autoantibodies are highly expressed in proximal tubules, which can also explain the wide range of kidney involvement in IPEX syndrome [9]. Furthermore, in our patient’s case, it is not clear whether non-lupus FHN was part of the clinical spectrum as a result of immune dysregulation, or was secondary to medications, infections or other concomitant insults. In this case, non-lupus FHN was treated as lupus nephritis and went into remission after the third cyclophosphamide pulse and remained in remission during azathioprine and later tacrolimus treatment. Similar to our results, high complete response rate was found in pediatric patients with non-lupus FHN [10].

## 3. What is the treatment and the prognosis of the disease?

**Fig. 1:**
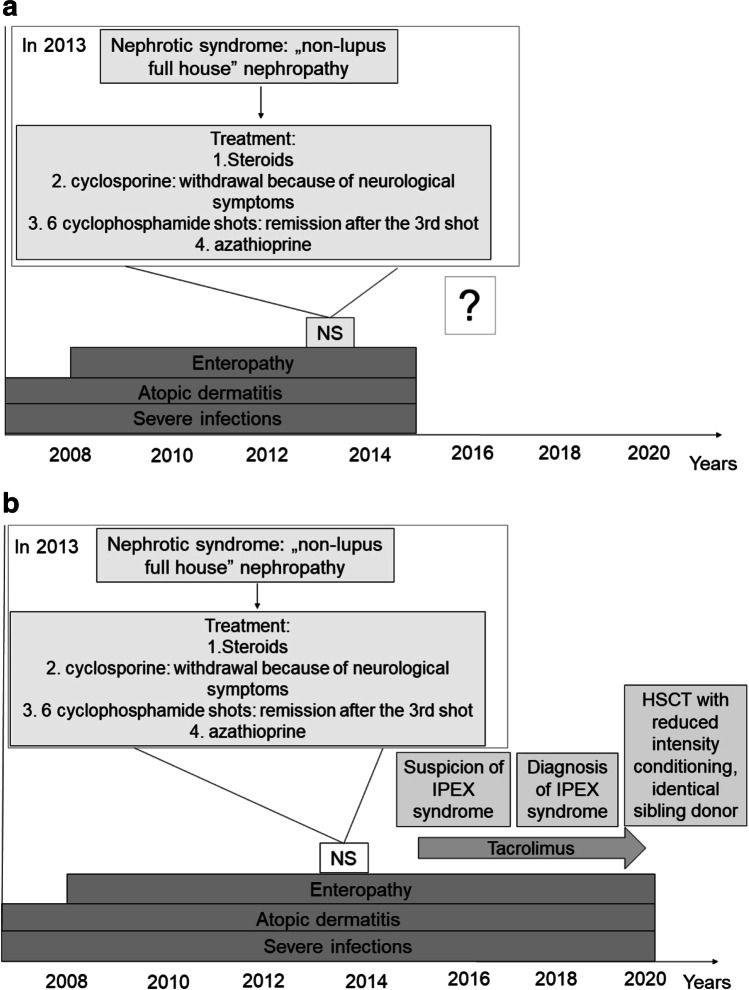
**a** Patient flow chart for the Questions part. NS indicates nephrotic syndrome. **b** Patient flow chart for the Answers part. HSCT indicates hematopoietic stem cell transplantation; IPEX, immunodysregulation, polyendocrinopathy, enteropathy, X-linked (IPEX) syndrome; NS, nephrotic syndrome. Tacrolimus was started as immunosuppressive therapy after the suspicion for IPEX syndrome and continued as graft-versus-host disease prophylaxis for 110 days after HSCT

Currently, the therapeutic approach to IPEX syndrome is not standardized; patient management is based on single-centre experiences. Immunosuppressive treatment (IST) is the treatment of choice; however, its role in controlling dysfunctional Treg cells is not clear [1]. Disease manifestations improve in half of the cases treated with IST alone [11]. Based on clinical signs and the results of flow cytometry, our patient was treated for IPEX-like syndrome after 2015 (Fig. 1). IST with steroids, cyclophosphamide, azathioprine and finally tacrolimus resulted in only partial control of the disease. The main reason for hematopoietic stem cell transplantation (HSCT) was uncontrolled autoimmune enteropathy, failure to thrive (body mass index (BMI) z-score –1.5), persisting skin symptoms and poor quality of life. HSCT is currently the only curative therapy for IPEX syndrome and can result in favourable outcome with improved quality of life [1, 11, 12]. In the present case, considering the non-malignant indication, reduced intensity HSCT conditioning regimen was used. Treatment-related toxicity was well tolerated and oral nutrition could be started. HSCT was performed with an identical female sibling donor and tacrolimus as graft-versus-host disease prophylaxis could be discontinued early, 4 months after HSCT.

## Patient outcome

One year after HSCT, at the age of 14, the patient lives without IST, has normal kidney function and blood pressure, no haematuria and proteinuria below 15 mg/m^2^/h. The patient tolerates normal enteral nutrition well, and moreover gained weight and height (BMI z-score 0.03) as well.

## Conclusion

In conclusion, severe immune dysregulation in IPEX syndrome can be an underlying cause in the pathomechanism of non-lupus full-house nephropathy, a rare cause of nephrotic syndrome in childhood. With combined IST, long-term remission of NS was achieved, HSCT was necessary to control the severe extrarenal clinical symptoms. In such cases of FHN, patients should be screened for primary immunodeficiency.

## Data Availability

Not applicable.
