# Correction to: Non-lupus full-house nephropathy—immune dysregulation as a rare cause of pediatric nephrotic syndrome: Answers

**DOI:** 10.1007/s00467-022-05437-0

**Published:** 2022-02-24

**Authors:** Orsolya Horváth, György S. Reusz, Veronika Goda, Kata Kelen, István Balogh, Magdolna Kardos, Krisztián Kállay, Áron Cseh, Attila J. Szabó, Gergely Kriván

**Affiliations:** 1 1St Department of Pediatrics, Semmelweis University, 53-54 Bókay János Street, Budapest, 1083 Hungary; 2 Pediatric Hematology and Stem Cell Transplantation Unit, Central Hospital of Southern Pest, National Institute of Hematology and Infectious Diseases, Budapest, Hungary; 3 Department of Laboratory Medicine, Division of Clinical Genetics, Department of Human Genetics, Faculty of Medicine, University of Debrecen, Debrecen, Hungary; 4 2Nd Department of Pathology, Semmelweis University, Budapest, Hungary

**Correction to: Pediatr Nephrol**

**https://doi.org/10.1007/s00467-021–05378-0**

Due to an unfortunate error, commentaries for the correction of the proof were rendered in the final version of this article. The commentary “The proof contains only the Answers part of the Clinical Quiz. Will the Questions part arrive in an another form?” was mistakenly left on the first heading of the article. The commentary “At the online publishing platform NKFI was marked incorrectly as National Kidney Foundation of Iowa, by mistake. This manuscript contains funding information correctly.” Was mistakenly left on the Funding section. These commentaries have been deleted and the article has been corrected. The publisher apologizes for this mistake.

